# CRISPR/Cas9 based blockade of IL-10 signaling impairs lipid and tissue homeostasis to accelerate atherosclerosis

**DOI:** 10.3389/fimmu.2022.999470

**Published:** 2022-08-30

**Authors:** Haozhe Shi, Jiabao Guo, Qiongyang Yu, Xinlin Hou, Lili Liu, Mingming Gao, Lili Wei, Ling Zhang, Wei Huang, Yuhui Wang, George Liu, Peter Tontonoz, Xunde Xian

**Affiliations:** ^1^ Institute of Cardiovascular Sciences and Key Laboratory of Molecular Cardiovascular Sciences, Ministry of Education, School of Basic Medical Sciences, Peking University, Beijing, China; ^2^ Department of Pediatrics, Peking University First Hospital, Beijing, China; ^3^ Laboratory of Lipid Metabolism, Institute of Basic Medicine, Hebei Medical University, Shijiazhuang, China; ^4^ School of Medicine, Shihezi University, Shihezi City, China; ^5^ Department of Pathology, University of California, Los Angeles, CA, United States; ^6^ Department of Biological Chemistry, University of California, Los Angeles, CA, United States; ^7^ Beijing Key Laboratory of Cardiovascular Receptors Research, Peking University Third Hospital, Beijing, China

**Keywords:** interleukin-10, atherosclerosis, gut microbiota, Syrian golden hamster, CRISPR/Cas9

## Abstract

Interleukin-10 (IL-10) is a widely recognized immunosuppressive factor. Although the concept that IL-10 executes an anti-inflammatory role is accepted, the relationship between IL-10 and atherosclerosis is still unclear, thus limiting the application of IL-10-based therapies for this disease. Emerging evidence suggests that IL-10 also plays a key role in energy metabolism and regulation of gut microbiota; however, whether IL-10 can affect atherosclerotic lesion development by integrating lipid and tissue homeostasis has not been investigated. In the present study, we developed a human-like hamster model deficient in IL-10 using CRISPR/Cas9 technology. Our results showed that loss of IL-10 changed the gut microbiota in hamsters on chow diet, leading to an increase in lipopolysaccharide (LPS) production and elevated concentration of LPS in plasma. These changes were associated with systemic inflammation, lipodystrophy, and dyslipidemia. Upon high cholesterol/high fat diet feeding, IL-10-deficient hamsters exhibited abnormal distribution of triglyceride and cholesterol in lipoprotein particles, impaired lipid transport in macrophages and aggravated atherosclerosis. These findings show that silencing IL-10 signaling in hamsters promotes atherosclerosis by affecting lipid and tissue homeostasis through a gut microbiota/adipose tissue/liver axis.

## Graphical Abstract

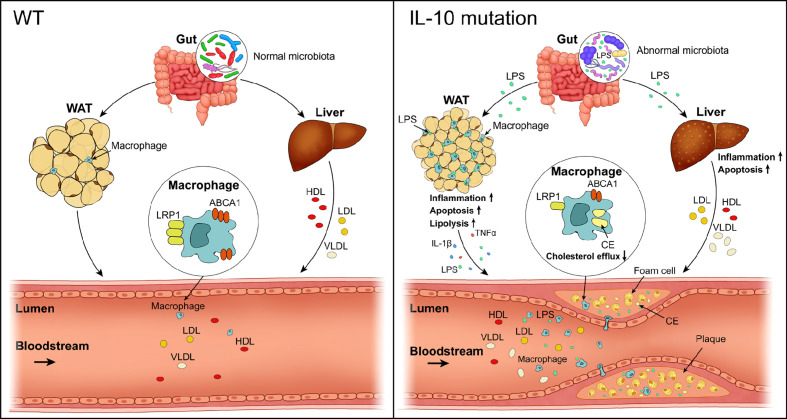

## Introduction

Inflammation and excess cholesterol accumulation in the vascular wall are important contributors to atherosclerosis. Increased low-density lipoprotein cholesterol (LDL-C) in the circulation enters the subendothelial space of the blood vessel wall through receptor-mediated pathways (1). After oxidative or other modifications, it promotes the infiltration and activation of inflammatory cells. Activated macrophages engulf cholesterol-rich lipoprotein particles to form foam cells, leading to a release of inflammatory cytokines, which in turn further accelerates intracellular cholesterol accumulation (2). It has been well documented that lowering blood cholesterol levels, especially LDL-C, through dietary intervention or medication such as statins, reduces the incidence of atherosclerosis (3). The existence of local inflammation is considered a potential risk factor for cardiovascular diseases (CVD), but the molecular mechanisms by which local inflammation promotes atherogenesis are not yet completely understood.

Interleukin-10, an important immunosuppressive factor, is mainly secreted by immune cells, including macrophages, NK-cells, B cells, and T cells. IL-10 inhibits the synthesis and secretion of proinflammatory cytokines in its target cells (4). However, the relationship between IL-10 and atherosclerosis is still under debate due to contradictory results from clinical and experimental studies. To our knowledge, mutations of IL-10 or its receptor in humans are primarily associated with inflammatory bowel disease (IBD) (5). Although it has been reported that IBD is closely related to the pathogenesis of atherosclerosis-related CVD (ASCVD) in some cases (6, 7), whether the link between IBD and CVD is attributable to the mutations of IL-10 or its receptor has not been carefully explored. Thus it has been difficult to define a protective effect of IL-10 on atherosclerosis and this has limited the potential application of IL-10 as a therapeutic approach for the treatment of ASCVD in clinical trials. Additionally, studies in different atherosclerosis-prone mouse models have also generated conflicting results (8–13), indicating that the net effects of IL-10 signaling on atherosclerosis remain to be fully clarified.

Recently evidence suggested that IL-10 is associated with the homeostasis of gut microbiota (14, 15). IL-10 synthesized and secreted by T or B cells upon the stimulation of microbiota has been reported to maintain the tissue homeostasis by regulating the steady state of microbiota. Moreover, a causal link between dysfunctional gut microbiota and lipid metabolism disorders has been reported independently (16, 17). These findings imply that IL-10 on atherosclerosis in both human and experimental mouse studies may not be solely due to effect of IL10 on inflammation. The molecular mechanisms by which IL-10 may modulate lipid metabolism to influence the development of atherosclerosis are largely unknown.

Given that Syrian golden hamsters possess metabolic features similar to humans (18–20), we generated an IL-10-deficient hamster model using CRISPR/Cas9 editing to investigate the role of IL-10 in lipid metabolism and atherosclerosis. Our results provide insight into the relationship between IL-10 and atherosclerosis and have potential therapeutic implications for the treatment of atherosclerosis.

## Materials and methods

### Animals

Wild type (WT) Syrian golden hamsters were purchased from vital river laboratory (Beijing, China). IL-10 mutant Syrian golden hamster model was generated by CRISPR/Cas9 gene editing technology in our laboratory. WT and mutant hamsters were maintained on a 14h light/10h dark cycle at 24°C. All the animals were fed a chow diet (CD) (20% protein and 4% fat; Beijing Ke’ao Company, Beijing, China) or a high-fat diet (HFD) containing 1% cholesterol and 15% fat with water ad libitum. At the endpoint of the experiments, animals were anesthetized with 3% pentobarbital sodium (45 mg/kg by intraperitoneal injection). All experiments were performed under the principle of experimental animal health (NIH released no.85Y231996 Revision) and approved by the laboratory animal ethics committee of Peking University (LA2010-059; 15 March 2010).

### Generation of IL-10 mutant (IL-10^MUT/MUT^) hamster model

The sgRNA was designed to target the exon 2 of IL-10 gene (NW_004801698) using Optimized CRISPR Design (http://crispr.mit.edu/). The specificity of the sgRNA target sites (TGAGTAGTATGTTGTCCAGCTGG) was analyzed according to the basic local alignment search tool (BLAST) applied to the Syrian golden hamster genome. The DNA template of sgRNA was amplified by PCR, and then transcribed to sgRNA by T7 polymerase (Megascript T7 Kit, Ambion, AMB13345) *in vitro*. The plasmid PXT7 carrying the humanized cas9 cDNA was linearized with XbaI digestion, which was used as Cas9 DNA template. Cas9 mRNA was transcribed with mMESSAGE mMACHINE T7 kit (Ambion, AM1344) *in vitro*. Cas9 mRNA and sgRNA were purified by phenol/chloroform extraction followed by isopropanol precipitation, which concentrations were adjusted to 500 ng/ul and 100 ng/ul with RNase-free water, respectively, and stored at -80°C for further experiments. Both microinjection and zygote treatment were performed under the condition of red light. M2 medium (Sigma, M7167) covered by mineral oil (Sigma, M5904) was used for injection. Cas9 mRNA (50 ng/μL) and sgRNA (20 ng/μL) were co-injected into the cytoplasm of zygotes. Afterward, zygotes were cultured with HECM-10 medium and 10% CO_2_ at 37.5°C for 30 min. Injected zygotes with normal morphology were transferred into surrogate hamsters that were naturally mated with males 1 day before (approximate 15–20 zygotes per oviduct). For genotyping, the genomic DNA extracted from the toes of the founder and his offspring was analyzed by PCR (IL-10 F: GCTTTCAGTGAAGTTTCCGTAT, IL-10 R: AAGTAACCCTAGAGGCAAGAAT).

### Analysis of plasma lipids and (apo)lipoproteins

Plasma was collected from WT and IL-10 mutant hamsters after 12h fasting. Total cholesterol (TC) and triglyceride (TG) levels were determined using the commercially enzymatic kits (Biosino Bio Technology & Science, Beijing, China). HDL-C level was measured with TC kit after precipitating ApoB-containing lipoprotein by 20% polyethylene glycol (PEG).

Plasma ApoA1, ApoB and ApoE were detected by Western blots. Briefly, 1ul of fresh plasma was mixed with buffer containing sodium dodecyl sulfate (SDS) and dithiothreitol (DTT), heated at 95°C for 10 min, and then subjected to 6% or 12% sodium dodecyl sulfate polyacrylamide gel (SDS-PAGE) gels for ApoB or ApoA1/ApoE, respectively. The following antibodies were used for immunoblotting: ApoA-I (NBP2-15429, Novus, USA, rabbit polyclonal IgG, 1:5000), ApoE (178479, Millipore, goat polyclonal IgG, 1:5000), and ApoB (178467, Millipore, goat polyclonal IgG, 1:5000).

### RNA isolation and quantitative real time PCR

Total RNA was extracted from different tissues by Trizol reagent (Transgen Biotech, China) and first-strand cDNA was generated by using a RT kit (Transgen Biotech, China). Quantitative real time PCR was performed using primers listed in Table S2. The reactions were performed using the Mx3000 Multiplex Quantitative PCR System for 40 cycles with denaturation at 94°C for 30s, annealing at 60°C for 45s, and extension at 72°C for 45s. Beta actin was used as internal control and all the data of gene expression were normalized to WT group.

### Fast protein liquid chromatography (FPLC)

Plasma lipoprotein profiles were analyzed by FPLC. The pooled plasma of each group (150 μL from 5-6 animals/group) was subjected to Tricorn high-performance Superose S-6 10/300GL column (Amersham Biosciences, Little Chalfont, Buckinghamshire, UK), followed by an elution with PBS at a constant flow rate of 0.8 mL/min. The TC and TG levels in each fraction (500 μL) were determined using the same commercial kits for TC and TG.

Three continuous fractions were mixed together with the buffer containing SDS and DTT, and heated at 95°C for 10 min. The contents of ApoA1, ApoB and ApoE were detected by immunoblotting according to the method described above.

### Plasma lipoprotein lipase (LPL) activity

Post-heparin plasma was collected 30 min after intraperitoneal injection of heparin (2000u/kg BW). A commercial LPL activity kit (ab204721, Abcam, USA) containing fluorescence labeled substrates and LPL activator was used to analyze plasma LPL activity. Briefly, 10 μL of post-heparin plasma from CD-fed WT or IL-10^MUT/MUT^ hamsters was incubated with 40 μL of phosphate buffer and 50 μL of diluted substrate at 37°C for 1h protected from light. The output at Ex/Em = 482/515 nm was measured every 10 min. LPL activity was present by FFA release (pmol/ml/min).

### Measurement of plasma lipopolysaccharide (LPS) and 8-isoprostane content

The level of plasma LPS were determined by commercial ELISA kit (LPS: E065061, 3AChem, China). 10 μL of fresh plasma was added to the pre-coated plate with primary antibody against LPS. The mixture was incubated at 37°C for 1h and then HRP-labeled anti-LPS antibody was applied to detect the level of LPS. The plate was washed extensively using washing buffer and the absorbance was measured at 450 nm. For the measurement of plasma 8-isoprostane, the commercial kit (No. 516351, Cayman Chemical, USA) was used. 50 μL of fresh plasma and 50 μL of Tracer was added to the pre-coated plate with primary antibody against 8-isoprostane. The plate was incubated at 4°C for 18h, and then was washed extensively using washing buffer and the absorbance was measured at 420 nm. The level of plasma 8-isoprostane could be calculated by the absorbance at 420 nm.

### Determination of plasma malondialdehyde (MDA) concentration

The plasma MDA concentration was measured with a commercial test kit (E2009, Applygen, China). Briefly, 100 μL of fresh plasma sample or serially diluted standard samples were mixed thoroughly with 300 μL of buffer containing sodium dodecyl sulfate and thiobarbituric. The mixture was incubated at 95°C for 30 min and then placed on ice for 5 min, followed by a centrifugation at 10, 000g for 10 min. 200 μL of supernatant was collected for fluorometric measurement using ex535 nm/em553 nm, which was converted to the concentrations according to the standard curve.

### Hepatic lipid measurements

100 mg of liver tissue was homogenized in 1ml cold phosphate buffer solution (PBS), and then 4 ml of chloroform/methanol (v:v = 2:1) was added. The mixture was vortexed for 2 min, and then allowed to stand for 20 min. After a centrifugation at 3000 rpm for 30 min, the chloroform layer was transferred to a new glass tube using a glass syringe and dried under nitrogen stream. Lipids were dissolved with 500 μL of 3% triton X-100, and the contents of cholesterol and triglyceride were measured according to the method described above.

### Glucose tolerance test (GTT)

After fasting for 12h, plasma from indicated groups on CD feeding was collected, which represented a sample at 0 min. Animals were then intraperitoneally injected with glucose solution (2g glucose/kg body weight), and blood samples were collected at 15, 30, 60, and 120 min after injection. Glucose levels were measured for each indicated time point.

### Insulin tolerance test (ITT)

After fasting for 12h, plasma from indicated groups on CD feeding was collected, which represented a sample at 0 min. Animals were then intraperitoneally injected with insulin (0.75U insulin/kg body weight), and blood samples were collected at 15, 30, 60, and 120 min after injection. Glucose levels were measured for each indicated time point.

### Very low density lipoprotein (VLDL) secretion assay

After fasting for 12h, plasma from indicated groups on CD feeding was collected, which represented a sample at 0 min. Animals were then intraperitoneally injected with 407 (CAS 9003-11-6, Sigma, Germany) at 1500mg/kg, and blood samples were collected at 30, 60, 120, 180 and 240 min after injection. TG concentration was measured for each time point. The slope represented VLDL secretion rate after linear regression.

### Test of blood biochemical parameters

20 μL of fresh blood samples were used for the measurement of the following biochemical parameters: the counts of WBC, MO, GR, LY, RBC, HCT, HGB, RDW, PLT, PCT, MPV and PDW.

### Pathological analysis

Hamsters at the indicated time points under different conditions were sacrificed and perfused with 20 ml of 0.01M PBS through the left ventricle. Liver, intestines, spleen, white fat, brown fat, brain, heart, and aorta were harvested and then fixed in 4% paraformaldehyde (PFA) overnight, followed by a transfer to 20% sucrose solution for dehydration. Then liver, intestines, spleen and heart were embedded in SAKURA Tissue-Tek^®^ O.C.T. Compound (Sakura Finetek USA, Inc., USA), and cryo-sectioned ​​after snap frozen with liquid nitrogen. The sections were stained with hematoxylin/eosin (HE) for morphological analysis, or oil red O (ORO) to analyze lipid deposition. White and brown fat were embedded in paraffin and sliced for HE staining.

To measure the expression of specific proteins in tissues, the following antibodies were used for immunohistochemical or immunofluorescence staining: LPS (1:200 rabbit polyclonal IgG, PAB526Ge01, Clond-Clone, China), CD68 (1:400 rabbit polyclonal IgG, BM3639, BOSTER, China). Sections were incubated with the blocking solution (PBS containing 10% goat serum) for 1 h at 37°C after antigen retrieval, and then incubated with the primary antibody overnight at 4°C. Then for immunohistochemical staining, sections were incubated with the appropriate biotinylated secondary antibodies (1:200, ABC Vectastain; Vector Laboratories, Burlingame, CA, USA) and visualized using 3.3′-diaminobenzidine (DAB; Vectastain, Vector Laboratories). For immunofluorescence staining, sections were incubated with biotinylated secondary anti-mice and anti-rabbit IgG (1:1000, E032220-01 and E032410-01, EarthOx, USA) and fluorescent mounting medium with DAPI (ZLI-9557, ZSGB-BIO, China) under dark conditions. Then the sections were observed and imaged by confocal microscopy (Leica SP8, Germany).

### Microbiota analysis

Fresh feces of 3-month old WT and IL-10^MUT/MUT^ hamsters on CD were collected and the total genome DNA was extracted. DNA concentration and purity were monitored on 1% agarose gels. 16S rRNA was amplified used specific primer. Sequencing libraries were generated using TruSeq^®^ DNA PCR-Free Sample Preparation Kit (Illumina, USA) and index codes were added. The library was sequenced on an Illumina NovaSeq platform and 250 bp paired-end reads were generated. Sequence analysis was performed by Uparse software (Uparse v7.0.1001, http://drive5.com/uparse/). Sequences with ≥97% similarity were assigned to the same OTUs QIIME software (Version 1.9.1) was used to evaluate the differences of samples in species complexity, including MetaStat, LEfSe, analysis of similarities (ANOSIM) and principal coordinates analysis (PCoA). The results were displayed by R software (Version 2.15.3).

### Statistical analysis

All data were presented as means ± SEM. Statistical analysis was performed using the Student’s t-test for the comparison between two groups) or two-way ANOVA for the comparison among multiple groups. p value <0.05 was considered a statistical significance.

## Results

### Generation of IL-10 mutant (IL-10MUT/MUT) hamster model

**Figure 1:**
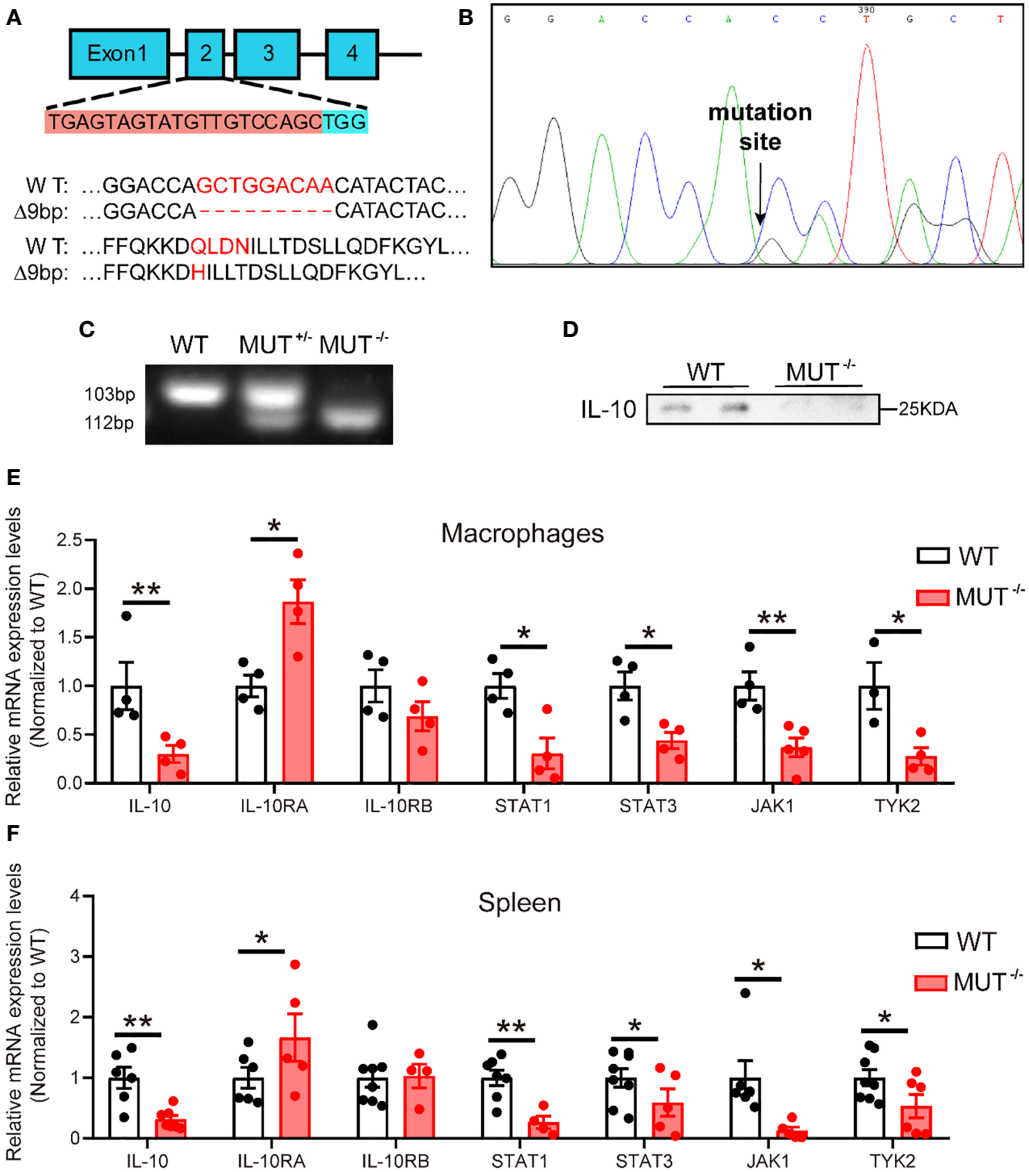
Generation and characterization of IL-10 mutant hamster model using CRISPR/Cas9 gene editing system. **(A)** Schematic of targeting the exon 2 of hamster IL-10 gene. The sgRNA and PAM sequences (TGG) are highlighted with orange and blue background, respectively. Mismatched nucleotide bases and amino acids are marked in red. **(B)** Sequencing of CRISPR/Cas9-edited IL-10 locus in Founder. Double peaks appeared at the mutation site. **(C)** Genotyping of F2 generations from wild type (WT), heterozygous (IL-10^WT/MUT^), and homozygous (IL-10^MUT/MUT^) IL-10 mutant hamsters. **(D)** Representative Western blot of plasma IL-10 from 3-month-old male WT and IL-10^MUT/MUT^ hamsters on chow diet. **(E** and **F)** mRNA expression levels of genes involved in IL-10 signaling pathway in macrophages **(E)** and spleen **(F)** were determined by real-time PCR (n=4~7/group). All data were expressed as means ± SEM, *p<0.05; **p<0.01.

In order to construct an IL-10 mutant hamster model, we designed sgRNA targeting exon 2 of the hamster IL-10 gene (**Figure 1A**). Zygotes microinjected with the designed sgRNA and Cas9 mRNA were transplanted into the fallopian tube of surrogate hamsters. Genomic DNA from the founders was extracted and sequenced. The sequencing data showed that one hamster pup (F1) displayed a 9 nt-deletion of IL-10 gene (**Figure 1B**), without any potential off-target sites (Table S1). Next, we genotyped the animals with F2 generation to determine whether this mutation of IL-10 gene was inheritable to offspring. PCR genotyping confirmed the presence of 112bp in wild-type (WT) and 103bp in homozygous mutant hamsters (IL10^MUT/MUT^) respectively, while heterozygous animals (IL10^WT/MUT^) showed two bands (**Figure 1C**). Protein analysis by Western blot revealed that plasma IL-10 levels of IL10^MUT/MUT^ hamsters were lower than in the WT group (**Figure 1D**). In addition, we found that the mRNA expression levels of IL-10 and the downstream targets of the IL-10 signaling pathway in macrophages (**Figure 1E**) and spleen (**Figure 1F**) were also markedly reduced. These findings validate our generation of an IL-10-mutant hamster model with impaired IL-10 signaling by CRISPR/Cas9 editing.

### Loss of IL-10 causes severe spontaneous systemic inflammation in hamsters

**Figure 2:**
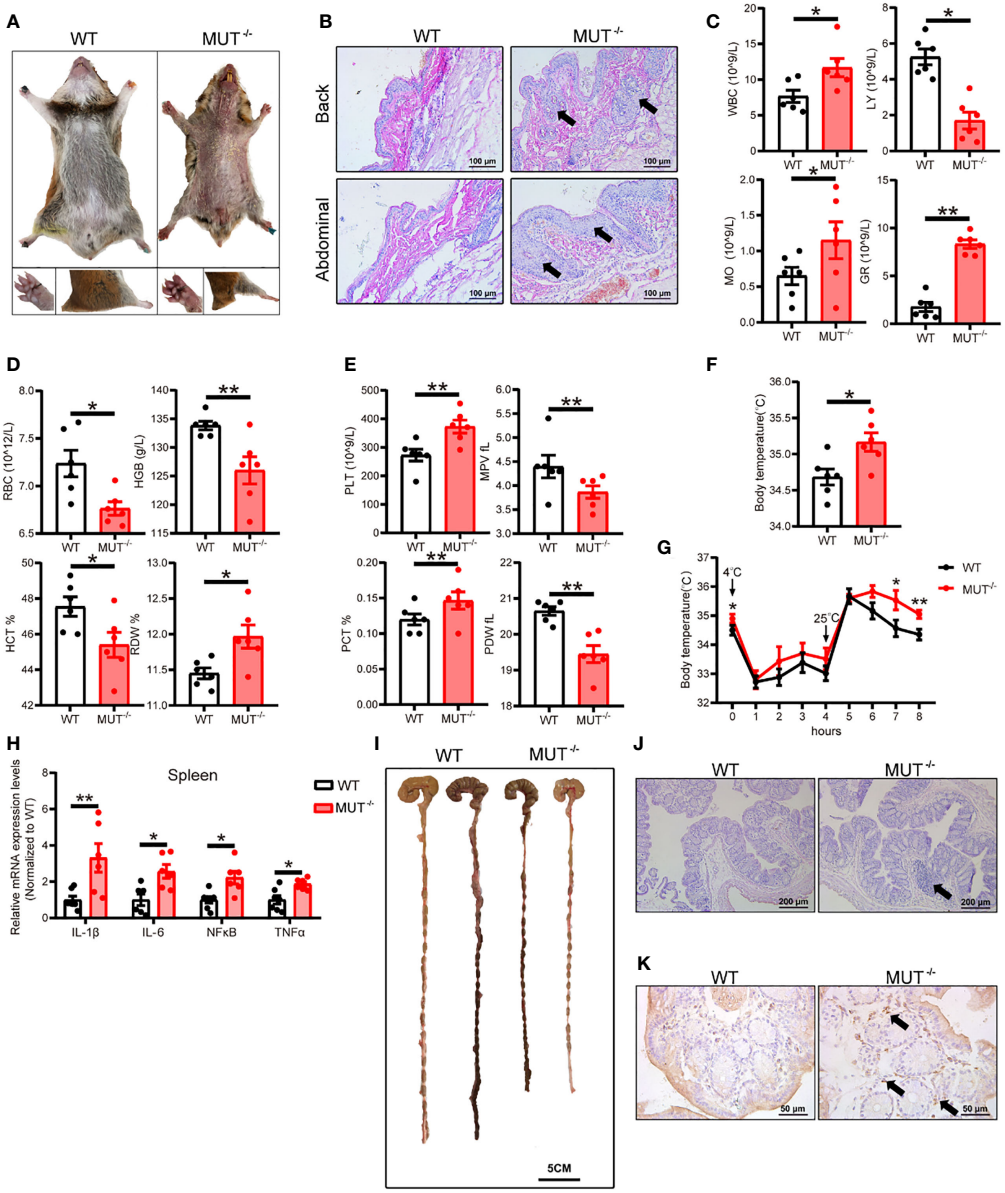
IL-10^MUT/MUT^ hamsters on a regular chow diet exhibited severe spontaneous systemic inflammation. **(A)** Representative images of 3-months old male WT and IL-10^MUT/MUT^ hamsters on chow diet. **(B)** Skin at the Back and abdominal areas of WT and IL-10^MUT/MUT^ hamsters was stained with HE. Bars: 100 μm; arrows indicate immune cell infiltration. **(C, D** and **E)** The blood biochemical characteristics of WT and IL-10^MUT/MUT^ hamsters (n=6/group). **(F)** Body temperature of WT and IL-10^MUT/MUT^ hamsters (n=6/group). **(G)** Body temperature curve of WT and IL-10^MUT/MUT^ hamsters exposed to different stimuli (n=6/group). **(H)** Expression levels of proinflammatory markers in spleen were determined by real-time PCR (n=6/group). **(I)** Representative images of colon tissue from WT and IL-10^MUT/MUT^ hamsters. Bars: 5 cm. **(J)** Morphological analysis of colons stained with HE for WT and IL-10^MUT/MUT^ hamsters. Bars: 500 μm; arrows indicate immune cell infiltration **(K)** Immunohistochemistry of CD68 in the colons from WT and IL-10^MUT/MUT^ hamsters. Bars: 50 μm; arrows indicate positive staining. All data were expressed as means ± SEM, *p<0.05; **p<0.01.

Considering that IL-10 is a major anti-inflammatory factor, we first investigated systemic inflammation IL10^MUT/MUT^ hamsters. 3-month-old male IL10^MUT/MUT^ hamsters maintained on chow diet (CD) showed obvious changes in external appearance compared to WT hamsters, including oral ulceration, abdominal hair loss, limb edema and reduced body weight (**Figure 2A**). H&E staining confirmed that hamsters with IL-10 deficiency developed spontaneous inflammatory skin lesions on the back and abdominal areas with inflammatory cell infiltration (**Figure 2B**). Complete blood tests revealed that compared to control group, the number of white blood cells (WBC), monocytes (MO) and granulocytes (GR) of IL10^MUT/MUT^ hamsters was increased; however, the counts of lymphocytes (LY) were largely decreased in mutant animals. These findings suggest a severe systemic inflammatory response in IL10^MUT/MUT^ hamsters (**Figure 2C**). In addition, red blood cell count (RBC), hematocrit (HCT) and hemoglobin levels (HGB) were reduced in IL10^MUT/MUT^ hamsters, and there was an increase in red blood cell volume distribution width (RDW), platelet count (PLT) and plateletocrit (PCT) (**Figure 2D**), and a reduction in mean platelet volume (MPV) and platelet distribution width (PDW) (**Figure 2E**).

Previous studies have shown a close relationship between inflammation and body temperature. Since an abnormal rise in body temperature can be an indicator of inflammation, we measured the body temperature of WT and IL10^MUT/MUT^ hamsters. As shown in **Figure 2F**, the body temperature of IL10^MUT/MUT^ hamsters was higher than WT hamsters. Upon cold stimulation followed by a switch to room temperature, the two genotypes showed no difference in body temperature under cold condition; however, the body temperature of IL10^MUT/MUT^ hamsters was still higher than WT hamsters after switching to room temperature (**Figure 2G**). These findings indicate that IL10^MUT/MUT^ hamsters had greater heat production than WT hamsters. To further confirm the inflammatory phenotype caused by IL-10 deficiency in hamsters, we determined the mRNA expression of proinflammatory markers in spleen, including IL-1β, IL-6, NFκB and TNFα. These proinflammatory cytokines were upregulated in IL10^MUT/MUT^ hamsters compared to WT hamsters (**Figure 2H**).

Since loss of function of IL-10 or its receptor leads to IBD in patients and mice, we explored whether IL10^MUT/MUT^ hamsters displayed the traits of IBD. We found that the colon length of IL10^MUT/MUT^ hamsters was shorter than that of WT hamsters, consistent with the presence of IBD (**Figure 2I**). Pathological analysis revealed an increase in the colon glands and the infiltration of inflammatory cells and macrophages (**Figures 2J and K**). Thus, IL10^MUT/MUT^ hamsters replicated the spontaneous IBD was observed in patients with dysfunctional mutations in IL-10 or its receptor.

### IL-10 deficiency causes severe white adipose tissue (WAT) loss in hamsters

**Figure 3:**
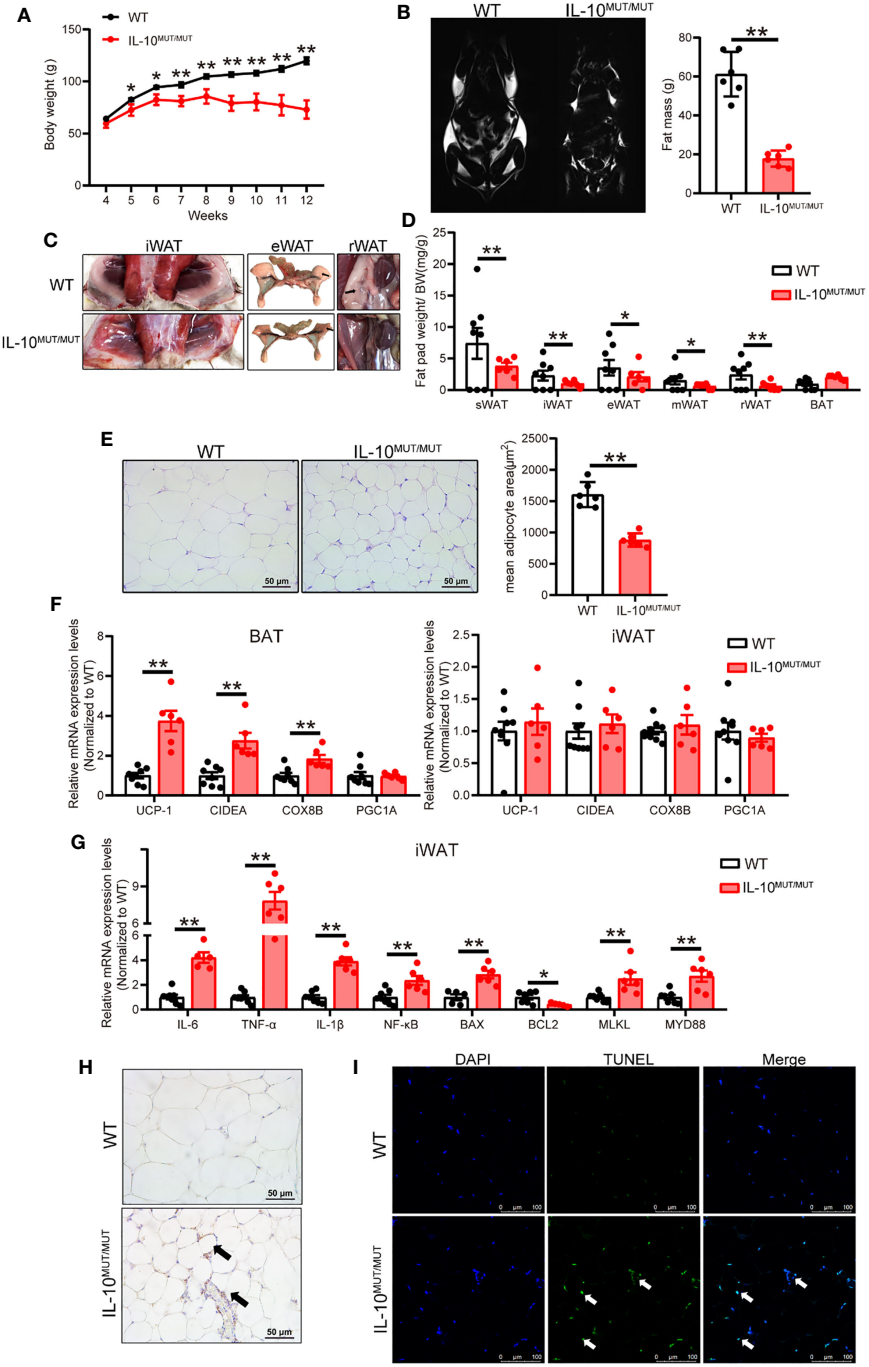
IL-10 deficiency caused severe white adipose tissue (WAT) dystrophy in chow-fed hamsters. **(A)** Body weight curve of WT and IL-10^MUT/MUT^ hamsters on chow diet (n=6/group). **(B)** Representative MRI images and total fat mass analyzed by MRI of 3-month-old male WT and IL-10^MUT/MUT^ hamsters (n=6/group). **(C)** Representative images of iWAT, eWAT and rWAT from WT and IL-10^MUT/MUT^ hamsters. Arrows indicate WAT. **(D)** Analysis of the ratio of fat weight and body weight for different types of adipose tissues from 3-months old WT and IL-10^MUT/MUT^ hamsters (n=6~8/group). **(E)** Representative images of iWAT (left) and quantification of adipocyte area (right) (n=6/group). **(F)** Expression levels of genes regulating thermogenesis in BAT (left) and iWAT (right) were determined by real-time PCR (n=5~7/group). **(G)** Expression levels of genes involved in inflammation and apoptosis in iWAT were determined by real-time PCR (n=5~7/group). **(H)** Immunohistochemistry of CD68 in the iWAT from WT and IL-10^MUT/MUT^ hamsters. Bars: 50 μm; arrows indicate positive staining. **(I)** TUNEL staining (green) in the iWAT from WT (top) and IL-10^MUT/MUT^ (bottom) hamsters. Nuclei were stained with DAPI (blue). Bars: 100 μm; white arrows indicate positive staining.All date were expressed as means ± SEM, *p<0.05; **p<0.01.

IL10^MUT/MUT^ hamsters showed decreased body weight and reduced fat mass compared to WT hamsters on CD (**Figures 3A, B**). The mass of multiple different subtypes of WAT, including subcutaneous WAT (sWAT), inguinal WAT (iWAT), epididymal WAT (eWAT), mesenteric WAT (mWAT) and retroperitoneal WAT (rWAT) was reduced in IL10^MUT/MUT^ hamsters, while the weight of brown adipose tissue (BAT) did not change (**Figures 3C, D**). Further analysis by H&E staining revealed that the size of adipocytes in WAT of IL10^MUT/MUT^ hamsters was reduced by ~50% (**Figure 3E**). Given the higher body temperature observed IL10^MUT/MUT^ hamsters, we determined the mRNA expression of genes regulating thermogenesis in adipose tissue, and found that the expression levels of CIDEA and UCP-1 in BAT were upregulated in IL10^MUT/MUT^ hamsters, but there was no difference in WAT gene expression between the two groups (**Figure 3F**). These observations suggest that activated thermogenesis in BAT might contribute to increased heat production in IL10^MUT/MUT^ hamsters.

It has been reported that inflammation plays an important role in the determination of fat cell size (21). We therefore analyzed the status of inflammation in WAT. In IL10^MUT/MUT^ hamsters, the mRNA expression level of proinflammatory factors was upregulated. Levels of genes involved in apoptosis, such as BAX, MLKL and MYD88, were also upregulated, but the level of BCL2, an anti-apoptotic gene, was decreased (**Figure 3G**). Immunohistochemical staining demonstrated an increase in CD68 protein and apoptosis stained by TUNEL in WAT of IL10^MUT/MUT^ hamsters (**Figures 3H, I**). These findings indicate that inflammation and apoptosis likely contribute to the reduced size of WAT in IL-10 mutant hamsters.

### IL-10 deficiency alters microbiome homeostasis and enhances LPS production

**Figure 4:**
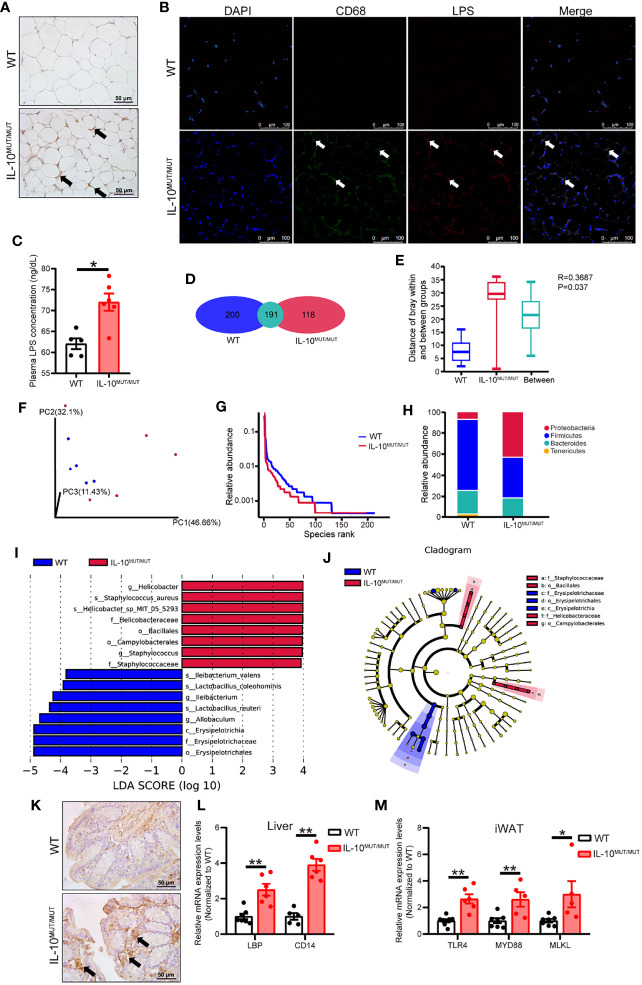
Loss of IL-10 disrupted the profile and products of gut microbiota in IL-10^MUT/MUT^ hamsters on chow diet. **(A)** Immunohistochemistry of LPS in the iWAT from WT and IL-10^MUT/MUT^ hamsters. Bars: 50 μm; arrows indicate positive staining. **(B)** Cryo-sections of iWAT from WT (top) and IL-10^MUT/MUT^ (bottom) hamsters were double-stained with CD68 (green) and LPS (red) antibodies. Nuclei were stained with DAPI (blue). Bars: 100 μm; white arrows indicate positive staining. **(C)** Plasma LPS levels were determined from WT and IL-10^MUT/MUT^ hamsters (n=5/group). **(D)** Analysis of the total number of OTUs present in WT hamsters (bule), IL-10^MUT/MUT^ hamsters (red) or overlap in both groups (Cyan). **(E)** Boxplots depicting differences in gut microbiota diversity in WT hamsters, IL-10^MUT/MUT^ hamsters and both the two groups. **(F)** Weighted Unifrac distance based analysis of PCoA. WT group was shown in blue, while the IL-10^MUT/MUT^ group was indicated in red. **(G)** Analysis of Rank abundance curves in WT and IL-10^MUT/MUT^ hamsters. The x-axis indicates the order number ranked by the OTUs abundance and Y-axis shows the relative abundance of OTUs. **(H)** The relative abundance of top 4 species at the levels of phylum in WT and IL-10^MUT/MUT^ hamsters. **(I)** LEfSe comparison of the gut microbiota between WT (bule) and IL-10^MUT/MUT^ (red) hamsters. LDA score >3 are shown. The length of the bar represents the LDA score. **(J)** LEfSe analysis of cladogram of significant changes at all taxonomic levels in WT and IL-10^MUT/MUT^ hamsters. The diameter of each circle is proportional to its abundance. **(K)** Immunohistochemistry of LPS in the colon from WT (left) and IL-10^MUT/MUT^ (right) hamsters. Bars: 50 μm; arrows indicate positive staining. **(L)** Expression levels of LBP and CD14 in liver were determined by real-time PCR (n=5/group). **(M)** Expression levels of TLR4, MYD88 and MLKL in iWAT were determined by real-time PCR (n=5/group). All date were expressed as means ± SEM, *p<0.05; **p<0.01.

LPS is a potent pro-inflammatory signal harbored by many gut microbes. We hypothesized that silencing IL-10 might disturb the homeostasis of gut microbiota, thus promoting the synthesis and secretion of LPS, and eventually leading to systemic inflammation. To verify this hypothesis, we first assayed LPS content in the WAT with overt morphological changes. Immunohistochemical staining confirmed that LPS levels were higher in IL-10^MUT/MUT^ hamsters than WT hamsters (**Figure 4A**). Immunofluorescence demonstrated that LPS in the WAT of IL10^MUT/MUT^ hamsters co-localized with the macrophage marker CD68 (**Figure 4B**), indicating that LPS was delivered to WAT from gut through macrophage-mediated transport. Similarly, we found that circulating LPS levels in IL10^MUT/MUT^ hamsters were markedly increased (**Figure 4C**), suggesting that gut function might be impaired due to IL-10 deficiency.

Next, when assessing gut function, we found that there were 191 OTUs shared in the gut microbiota of the two genotypes, while there were 200 and 118 unique OTUs in WT and IL10^MUT/MUT^ hamsters, respectively (**Figure 4D**), with a significant difference between the two groups when analyzed by Anoism (R=0.3687 and P=0.037, **Figure 4E**). Meanwhile, we found a separation between the genotypes using Weighted Unifrac distance-based PCoA analysis (**Figure 4F**) and a reduction in the species richness in IL10^MUT/MUT^ hamsters relative to control animals (**Figure 4G**). Furthermore, IL10^MUT/MUT^ hamsters had a greatly increased content of proteobacteria in the gut, while the counts of firmicutes and bacteroidetes, two types of beneficial bacteria, were reduced (**Figure 4H**). As expected, LefSe analysis showed that IL10^MUT/MUT^ hamsters exhibited an increase in several types of pathogenic bacteria, such as *helicobacter* and *staphylococcus*, but a decrease in the probiotic bacteria that exert protective effects on the gut barrier, such as *lactobacillus*, *allobaculum*, *erypelotrichia* (**Figures 4I, J**). These changes likely contribute to the destruction of the gut barrier and the increased secretion of gut-derived LPS into plasma. To verify this point, we determined the signaling pathways related to LPS responses in various tissues. Immunohistochemical staining of the colon showed an increase in LPS levels in IL10^MUT/MUT^ hamsters (**Figure 4K**). Hepatic mRNA expression of LBP and CD14 was upregulated in IL10^MUT/MUT^ hamsters (**Figure 4L**), indicating that the pathway regulating LPS transport was activated. Moreover, we found that the mRNA expression levels of TLR4, MYD88 and MLKL in WAT were markedly increased, (**Figure 4M**), suggesting that LPS signaling was activated in WAT. These findings support enhanced transport of LPS to WAT to activate the TLR4-mediated apoptotic pathway, thus triggering abnormal apoptosis in the WAT.

### Loss of IL-10 signaling elicits abnormal lipid and glucose metabolism in hamsters on chow diet

**Figure 5:**
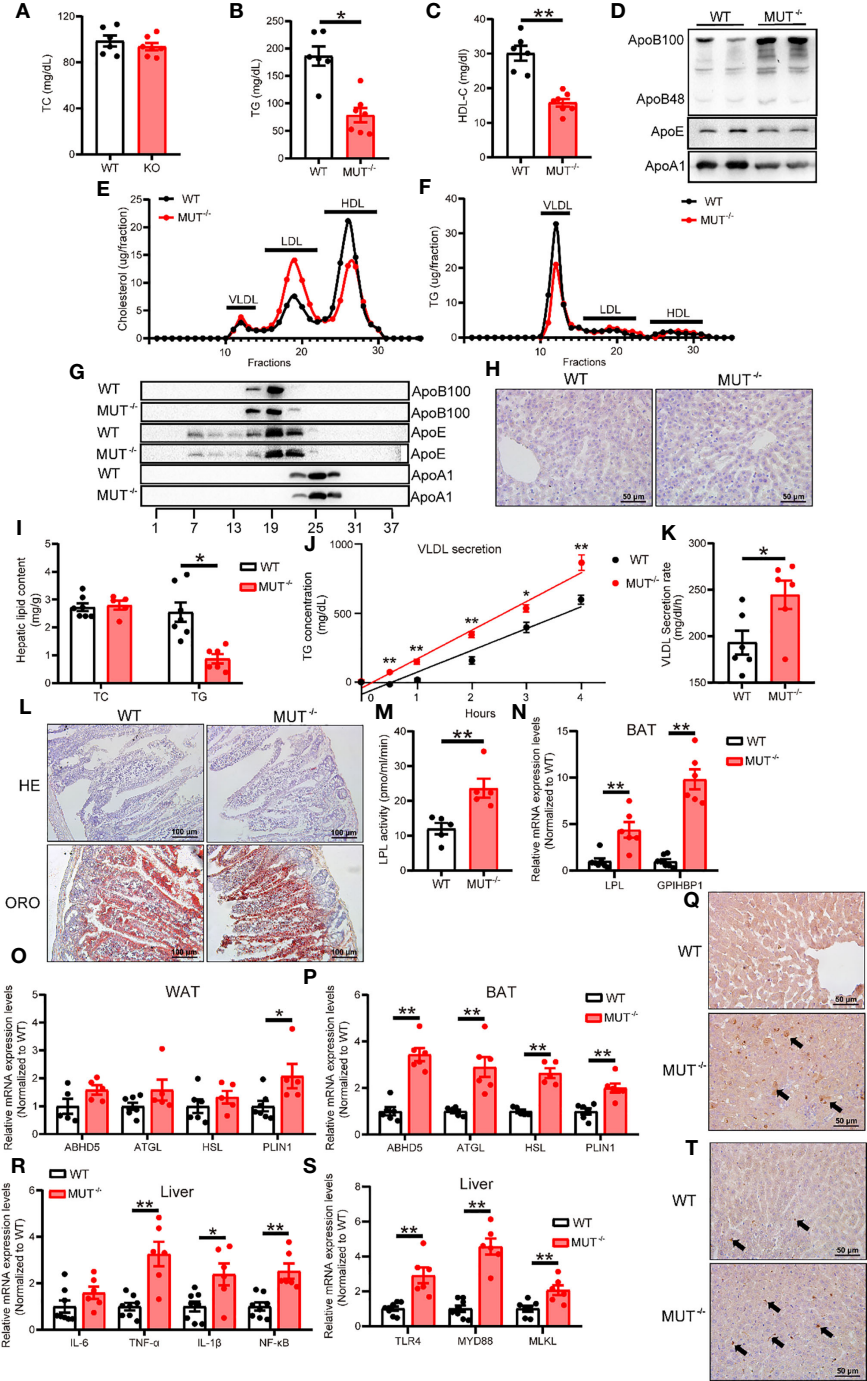
Chow-fed IL-10^MUT/MUT^ hamsters displayed abnormal lipid metabolism. **(A, B** and **C)** Determination of plasma TC **(A)**, TG **(B)** and HDL-C **(C)** from 3-month old WT and IL-10^MUT/MUT^ hamsters on chow diet after overnight fasting (n=6~7/group). **(D)** Representative Western blots of plasma ApoB, ApoE and ApoA1 from WT and IL-10^MUT/MUT^ hamsters. **(E, F)** Pooled plasma from the two groups were analyzed by FPLC. Triglyceride **(E)** and cholesterol **(F)** contents in different fractions of pooled plasma from WT and IL-10^MUT/MUT^ hamsters were measured (n = 6/group). **(G)** Representative Western blots of ApoB, ApoE and ApoA1 in different fractions of pooled plasma from WT and IL-10^MUT/MUT^ hamsters as described in **Figures 5E, F**. **(H)** Cryo-sectionings of liver tissues were stained with oil red O. Bars: 50 μm. **(I)** Hepatic TC and TG contents were measured and normalized to liver weight (n=6~7/group). **(J, K)** VLDL secretion was analyzed in hamsters after intraperitoneal injection P-407 (1500 mg/kg, n=6/group). **(L)** HE and Oil red O stainings of intestinal tissues of WT and IL-10^MUT/MUT^ hamsters 4 h after oral gavage of olive oil (10 ml/kg body weight). **(M)** Measurement of plasma LPL activities were in WT and IL-10^MUT/MUT^ hamsters. **(N)** Expression levels of LPL and GPIHBP1 in BAT were determined by real-time PCR (n=5/group). **(O, P)** Expression levels of genes involved in lipolysis in WAT **(O)** and BAT **(P)** were determined by real-time PCR (n=5/group). **(Q)** Immunohistochemistry of LPS in the liver from WT (top) and IL-10^MUT/MUT^ (bottom) hamsters. Bars: 50 μm; arrows indicate positive staining. **(R)** Expression levels of TLR4, MYD88 and MLKL in liver were determined by real-time PCR (n=6~7/group). **(S)** Expression levels of inflammatory factors in liver were determined by real-time PCR (n=6/group). **(T)** Immunohistochemistry of CD68 in the liver from WT (top) and IL-10^MUT/MUT^ (bottom) hamsters. Bars: 50 μm; arrows indicate positive staining. All date were expressed as means ± SEM, *p<0.05; **p<0.01.

Considerable evidence supports a link between inflammation and the metabolic syndrome; however, the influence of IL-10 deficiency on lipid and glucose homeostasis is incompletely understood. We analyzed the plasma lipids in CD-fed animals at 3 months of age. We found that IL10^MUT/MUT^ hamsters displayed reduced TG and HDL-C levels without changes in TC concentration when compared to control animals (**Figures 5A–C**). Western blots showed that plasma ApoB100, ApoB48 levels were increased, but ApoA1 levels were reduced, in IL10^MUT/MUT^ hamsters. Levels of ApoE were not different (**Figure 5D**). FPLC analysis revealed that the peak of LDL-C was markedly increased, whereas HDL-C fractions were decreased in IL10^MUT/MUT^ hamsters (**Figure 5E**). There was a reduction in TG carried on VLDL particles (**Figure 5F**), consistent with the findings of apolipoprotein distribution (**Figures 5G–I**). In addition, although the loss of IL-10 signaling did not influence the expression of cholesterol synthesis-related genes in the liver, the expression of key genes involved in reverse cholesterol transport (RCT) such, as LCAT and ABCA1 were reduced (**Figure S1A**). There was also as well as a reduction in genes regulating TG synthesis in IL10-deficient animals (**Figure S1B**).

To explore the potential mechanisms by which lack of IL-10 caused reduced TG levels in IL10^MUT/MUT^ hamsters, we injected P407, an LPL inhibitor, to examine hepatic VLDL secretion. IL10^MUT/MUT^ hamsters exhibited a higher VLDL secretion rate (**Figures 5J, K**). We observed no difference in lipid content of intestinal epithelial cells after oral fat load (**Figure 5L**); however, plasma LPL activity was greatly increased (**Figure 5M**), indicating that the reduction in TG may be attributable to enhanced LPL-mediated lipolysis of TG-rich lipoproteins in IL10^MUT/MUT^ hamsters. We measured expression of genes regulating TG metabolism in both BAT and WAT and found that the mRNA expression of both LPL and GPIHBP1 was upregulated in BAT (**Figure 5N**). The expression of genes required for TG lipolysis in BAT such as ABHD5, ATGL, HSL and PLIN1 were also upregulated (**Figures 5O, P**).

Recently, the gut-liver axis has been shown to play an important role in the regulation of lipid metabolism. We investigated whether gut-derived LPS might contribute to abnormal VLDL secretion by the liver in the absence of IL-10. Immunohistochemical staining showed that IL10^MUT/MUT^ hamsters exhibited more LPS in the liver compared to WT (**Figure 5Q**), and the expression of TLR4 and its downstream genes in the liver was increased (**Figures 5R, S**). We also found obvious macrophage infiltration (**Figure 5T**).

A previous study reported a relationship between IL-10 and insulin sensitivity in mice, indicating that IL-10 might regulate glucose metabolism. We found that the fasting blood glucose levels of IL10^MUT/MUT^ hamsters on CD were lower than WT hamsters (**Figure S2A**), but that glucose tolerance (**Figure S2B**) and insulin tolerance (**Figure S2C**) were reduced. Western blot analysis showed that the protein levels of both AKT and pAKT in the liver of IL10^MUT/MUT^ hamsters were increased (**Figure S2D**), accompanied by upregulated expression of genes involved in hepatic gluconeogenesis, such as G6PC, GCK, PFKM and SDHA (**Figure S2E**). These finding suggest that IL-10 deficiency resulted in abnormal glucose metabolism in hamsters.

### High-fat diet aggravated abnormal plasma lipid profiles in IL10^MUT/MUT^ hamsters

**Figure 6:**
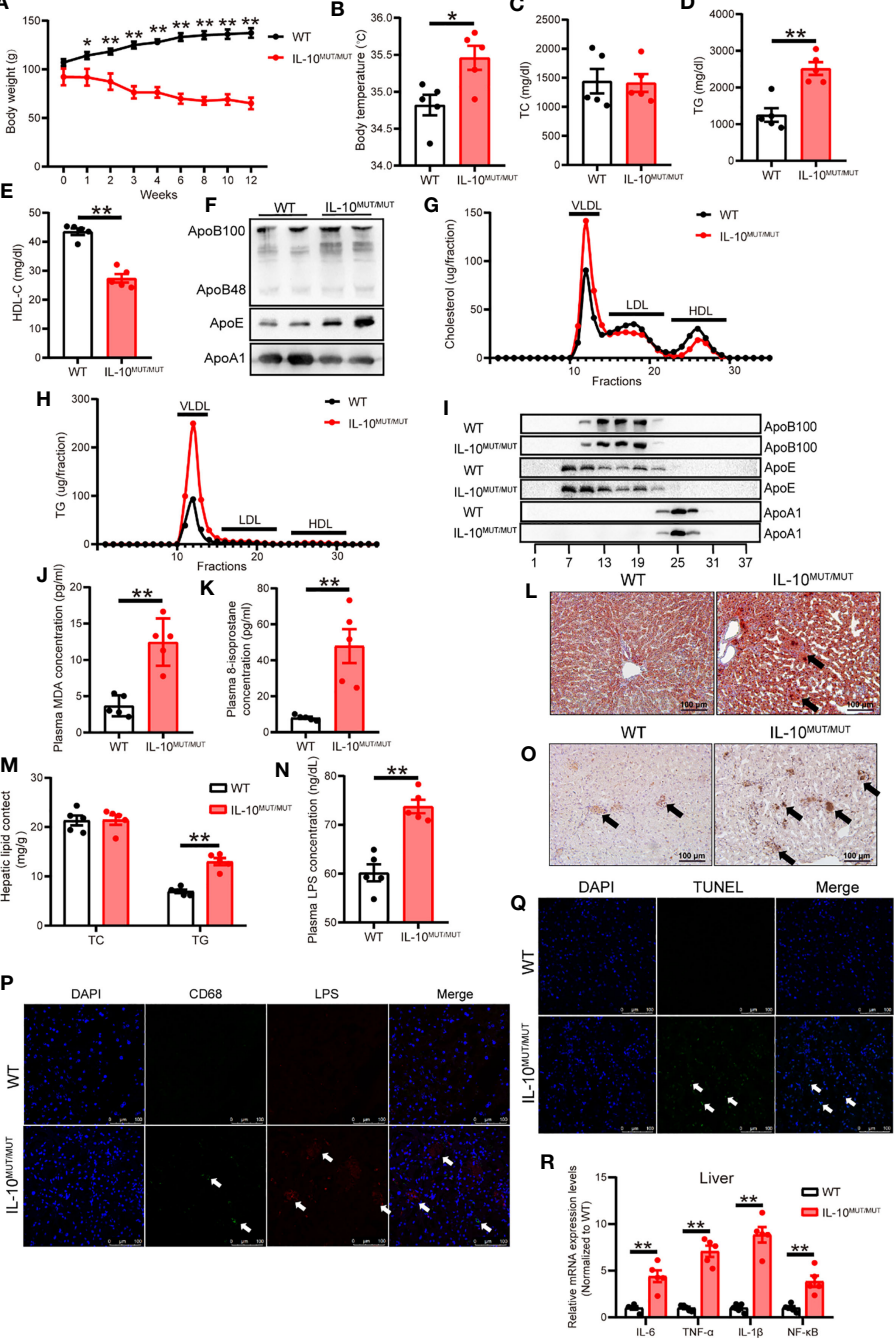
Dyslipidemia was aggravated in IL-10^MUT/MUT^ hamsters on HFD. **(A)** Body weight curve of WT and IL-10^MUT/MUT^ hamsters on HFD for 12 weeks (n=5/group). **(B)** Body temperature of WT and IL-10^MUT/MUT^ hamsters on HFD (n=5/group). **(C, D** and **E)** Plasma TC **(C)**, TG **(D)** and HDL-C **(E)** from WT and IL-10^MUT/MUT^ hamsters on HFD (n=5/group). **(F)** Representative Western blots of plasma ApoB, ApoE and ApoA1 from WT and IL-10^MUT/MUT^ hamsters on HFD. **(G, H)** Pooled plasma from the two groups were analyzed by FPLC. Triglyceride **(E)** and cholesterol **(F)** contents in different fractions of pooled plasma from WT and IL-10^MUT/MUT^ hamsters on HFD (n = 6/group). **(I)** Representative Western blots of ApoB (top), ApoE (middle) and ApoA1 (bottom) in different fractions. **(J, K)** Plasma MDA **(J)** and 8-isoprostane **(K)** concentration from WT and IL-10^MUT/MUT^ hamsters on HFD (n=5/group). **(L)** Oil red O stainings of liver tissue of WT and IL-10^MUT/MUT^ hamsters on HFD. Bars: 50 μm. Arrows indicate lipid accumulation. **(M)** Hepatic TC and TG contents were measured in HFD-fed animals. (n=6~7/group). **(N)** Plasma LPS levels from WT and IL-10^MUT/MUT^ hamsters on HFD (n=5/group). All values are means ± SEM, **, p<0.01. **(O)** Immunohistochemistry of CD68 in the liver from WT (left) and IL-10^MUT/MUT^ (right) hamsters on HFD. Bars: 50 μm; arrows indicate positive stainings. **(P)** Cryo-sections of liver from WT (top) and IL-10^MUT/MUT^ (bottom) hamsters on HFD were double-stained with CD68 (green) and LPS (red) antibodies. Nuclei were stained with DAPI (blue). Bars: 100 μm; white arrows indicate positive stainings. **(Q)** TUNEL staining (green) in the iWAT from WT (top) and IL-10^MUT/MUT^ (bottom) hamsters on HFD. Nuclei were stained with DAPI (blue). Bars: 100 μm; white arrows indicate positive stainings. **(R)** Expression levels of inflammatory factors in liver were determined by real-time PCR (n=5/group). All values are means ± SEM, **p<0.01. All date were expressed as means ± SEM, *p<0.05; **p<0.01.

To investigate whether high-fat diet (HFD) further aggravated abnormal lipid metabolism in the setting of inflammation caused by IL-10 deficiency, WT and IL10^MUT/MUT^ hamsters were fed a HFD containing 1.5% cholesterol and 15% fat for 16 weeks. Similar to what we observed in the animals on CD feeding, IL10^MUT/MUT^ hamsters on HFD showed less weight gain (**Figure 6A**) and higher body temperature (**Figure 6B**). Compared with WT hamsters, plasma HDL-C levels were still lower without a change in TC levels (**Figures 6C, D**), but the concentration of plasma TG of IL10^MUT/MUT^ hamsters was unexpectedly increased (**Figure 6E**). Western blots showed that plasma ApoB48 and ApoE levels were markedly increased, but APOA1 was reduced in IL10^MUT/MUT^ hamsters, with no obvious changes in ApoB100 (**Figure 6F**). Lipid profiles analyzed by FPLC demonstrated that cholesterol content was increased in the VLDL fraction and reduced in the HDL fractions in IL10^MUT/MUT^ hamsters (**Figure 6G**). Moreover, the TG concentration in the VLDL fraction was increased in mutant animals (**Figure 6H**). Consistent with the observations from plasma samples, Western blots of FPLC fractions revealed that the amount of ApoE carried on TG-rich lipoproteins was increased and the ApoA1 in HDL fractions was reduced in IL10^MUT/MUT^ hamsters (**Figure 6I**). Additionally, we found that the plasma levels of MDA (**Figure 6J**) and 8-isoprostane (**Figure 6K**) in IL10^MUT/MUT^ hamsters were increased, indicating accelerated lipid peroxidation.

Since dyslipidemia is associated with liver disease, we analyzed the lipid content of the liver in HFD-fed hamsters. There was increased TG content in liver of IL10^MUT/MUT^ hamsters, and there were more areas degeneration (**Figure 6L**), showing more, but not cholesterol (**Figure 6M**).

To study the effects of HFD on LPS production and transport through gut-liver axis, we investigated the levels of LPS in plasma and liver tissue and found that LPS levels were increased in both plasma and liver (**Figures 6N–P**). Furthermore, TUNEL staining and PCR analysis showed that apoptotic signals and inflammation were increased in the liver of IL10^MUT/MUT^ hamsters (**Figures 6Q, R**).

### IL-10 deficiency accelerates the development of atherosclerosis on HFD

**Figure 7:**
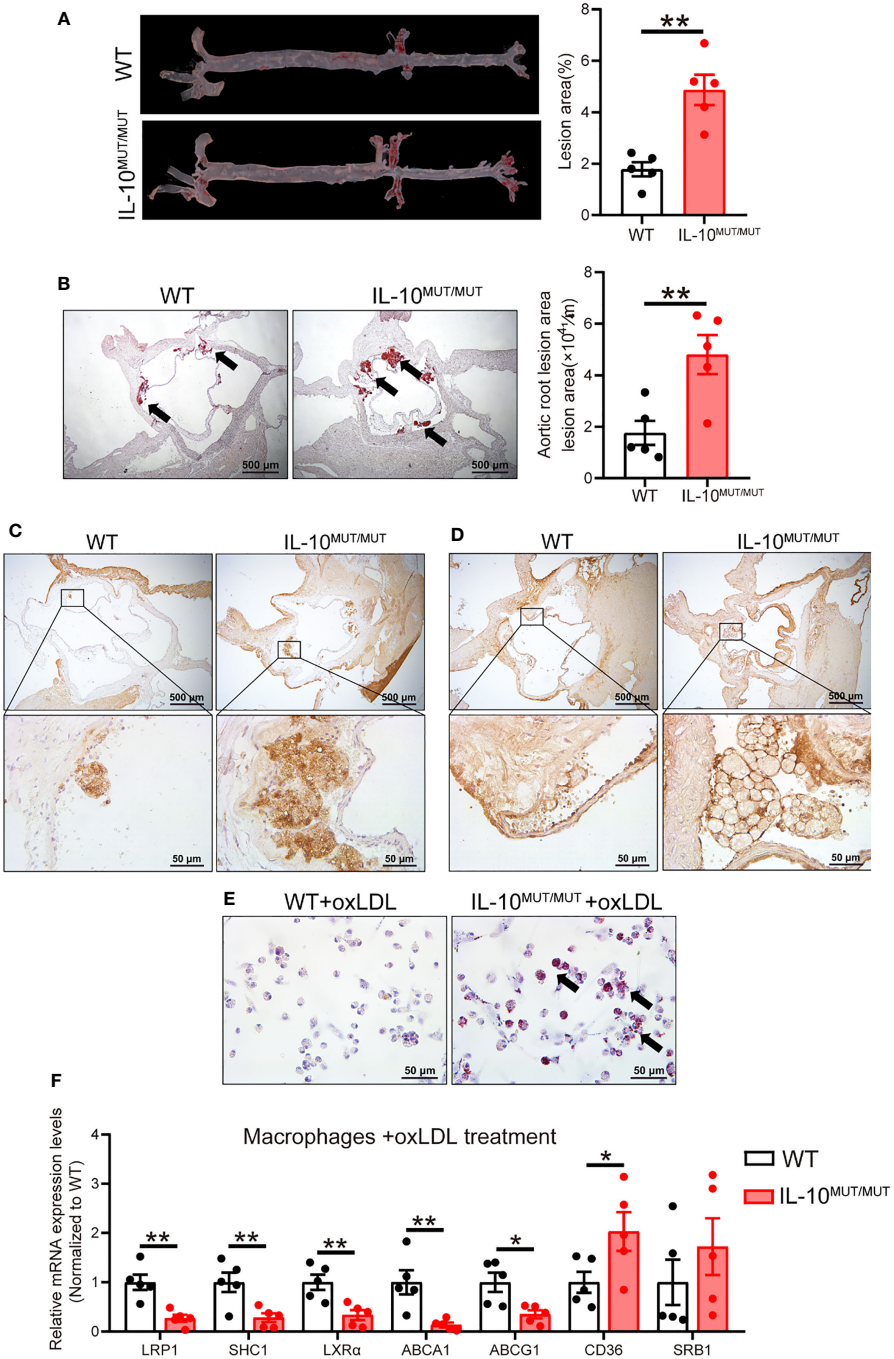
IL-10 deficiency accelerated atherosclerotic development in HFD-fed hamsters. **(A, B)** Analysis of atherosclerotic lesions in whole aorta **(A)** and sectioned aortic roots **(B)**. Representative images were shown in left panel and quantification was analyzed in right panel (n=5/group). Arrows indicate ORO-positive lesions. **(C, D)** Immunohistochemistry of CD68 **(C)** and LPS **(D)** in aortic roots from WT and IL-10^MUT/MUT^ hamsters on HFD. Bars: 500 μm (top) and 50 μm (bottom). **(E)** Representative photomicrographs of macrophages stained with ORO. Bars: 50 μm. Arrows indicate ORO positive cells. **(F)** Expression levels of genes involved in lipid uptake and RCT were determined by real-time PCR (n=5/group). All date were expressed as means ± SEM, *p<0.05; **p<0.01.

Since dyslipidemia can lead to atherosclerosis, we performed oil red O (ORO) staining to analyze atherosclerotic plaques. Atherosclerotic lesion area in both whole aorta (**Figure 7A**) and the aortic root (**Figure 7B**) was increased in IL10^MUT/MUT^ hamsters with accumulated CD68 (**Figure 7C**) and LPS (**Figure 7D**) after 16-week HFD feeding. To better understand the mechanism by which macrophages contributed to the atherosclerotic plaques in IL10^MUT/MUT^ hamsters, we incubated peritoneal macrophages isolated from WT and IL10^MUT/MUT^ hamsters with oxLDL. ORO staining showed that the intracellular lipid accumulation in macrophages of IL10^MUT/MUT^ hamsters was higher than WT hamsters (**Figure 7E**). Since LRP1 has been shown to integrate macrophage inflammation and cholesterol homeostasis (21), we investigated whether IL-10 deficiency led to an impairment of the LRP1 signaling pathway in our model. The mRNA expression of genes involved in cholesterol efflux regulated by LRP1 were reduced in macrophages of IL10^MUT/MUT^ hamsters, including SHC1, LXRα, ABCA1, and ABCG1. The expression of CD36, which is involved in lipid uptake, was increased (**Figure 7F**). These findings suggest that loss of IL-10 increased lipid uptake and impaired reverse cholesterol transport (RCT), leading to lipid accumulation and accelerated atherosclerosis.

### Recombinant IL-10 alleviates the phenotype of IL10^MUT/MUT^ hamsters

**Figure 8:**
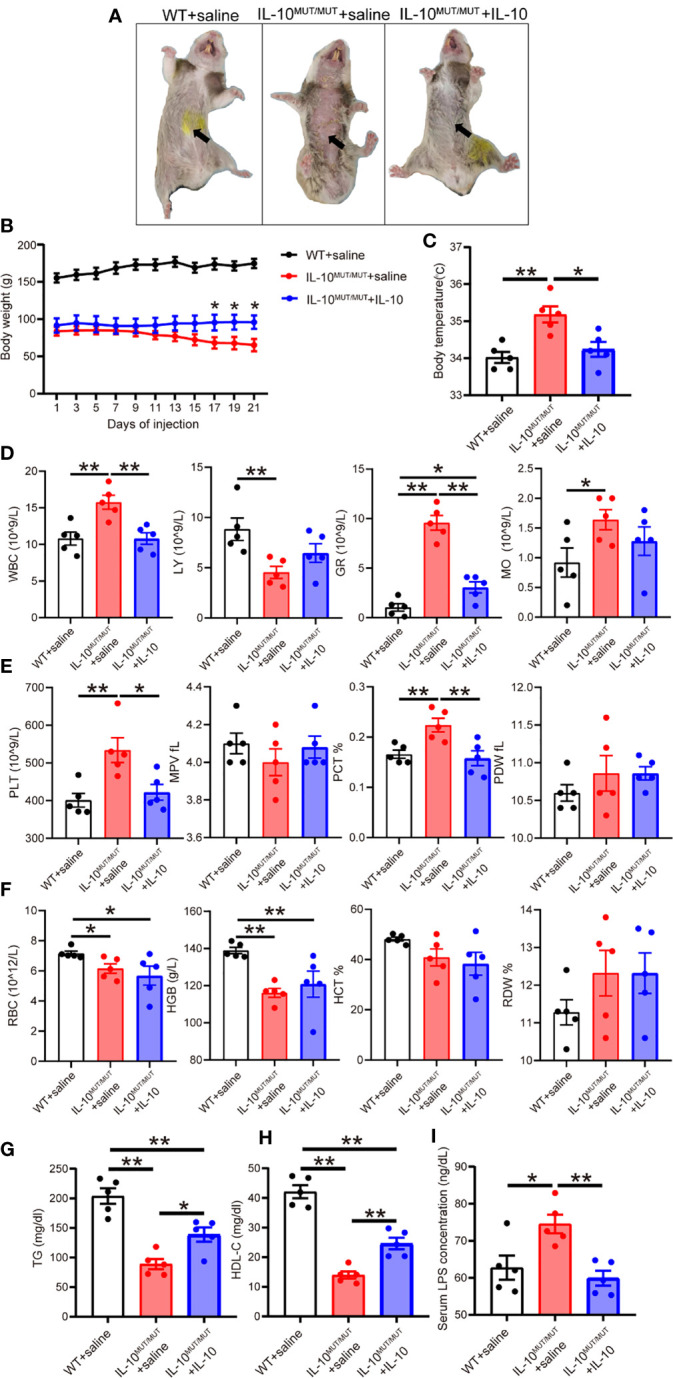
Short-term injection of rhIL-10 corrected the abnormal phenotypes of IL-10^MUT/MUT^ hamsters on chow diet. **(A)** Representative images of WT hamsters injected with saline (WT + saline), IL-10^MUT/MUT^ hamsters injected with saline (IL-10^MUT/MUT^ + saline) and IL-10^MUT/MUT^ hamsters intraperitoneally injected with rhIL-10 for 21 days (1 μg/kg/day, IL-10^MUT/MUT^ + IL-10). **(B)** Body weight curve of WT + saline, IL-10^MUT/MUT^ + saline and IL-10^MUT/MUT^ + IL-10 hamsters (n=5/group). **(C)** Body temperature of WT + saline, IL-10^MUT/MUT^ + saline and IL-10^MUT/MUT^ + IL-10 hamsters on day 21 after injection (n=5/group). **(D, E** and **F)** The blood biochemical characteristics of WT + saline, IL-10^MUT/MUT^ + saline and IL-10^MUT/MUT^ + IL-10 hamsters on day 21 after rhIL-10 injection (n=5/group). **(G, H)** Plasma TG **(G)** and HDL-C **(H)** from WT + saline, IL-10^MUT/MUT^ + saline and IL-10^MUT/MUT^ + IL-10 hamsters on day 21 after injection (n=5/group). **(I)** Plasma LPS level from WT + saline, IL-10^MUT/MUT^ + saline and IL-10^MUT/MUT^ + IL-10 hamsters on day 21 after injection (n=5/group). All date were expressed as means ± SEM, *p<0.05; **p<0.01.

We postulated that restoring IL-10 levels by replacement therapy should correct the phenotypes observed in IL10^MUT/MUT^ hamsters. To verify this hypothesis, we administered 8-week-old IL10^MUT/MUT^ hamsters 1 μg/kg human recombinant IL-10 (rhIL-10) daily for 3 weeks. Skin inflammation in IL10^MUT/MUT^ hamsters receiving rhIL-10 was obviously reduced compared with saline controls (**Figure 8A**). The body weight of the rhIL-10 group was also increased (**Figure 8B**) and the body temperature of rhIL-10 group was normalized (**Figure 8C**). Complete blood tests confirmed that IL-10 replacement completely corrected the inflammatory status and the number of WBC, GR, MO and LY in rhIL-10 hamsters (**Figure 8D**). Although the platelet-related indexes of rhIL-10 group were lower than the saline-treated group, (**Figure 8E**), there was no effect on RBC or HGB levels (**Figure 8F**). Finally, we assessed the effects of rhIL-10 therapy on lipid metabolism and found increased plasma TG and HDL-C levels (**Figures 8G, H**). rhIL-10 also normalized plasma LPS (**Figure 8I**).

## Discussion

Although the anti-inflammatory role of IL-10 is well documented, the relationship between IL-10, inflammation and lipid metabolism is still not completely understood. In particular, conflicting results have been reported on the influence of IL-10 on atherosclerosis in experimental mouse models. In the present study we developed a mutant hamster model lacking IL-10 using CRISPR/Cas9 gene editing. Our results showed that loss of IL-10 signaling impaired the homeostasis of gut microbiota, leading to release of LPS into the circulation. LPS in turn caused dystrophy of WAT by increasing LPL-mediated lipolysis and apoptosis of adipocytes, and abnormal lipid metabolism. IL10^MUT/MUT^ hamsters showed increased VLDL secretion by the liver, reduced hepatic　expression of LCAT and ABCA1, and blockade of LRP1-mediated cholesterol efflux from macrophages. Ultimately, all of these changes were associated with accelerated atherosclerotic development on a lipid-rich diet.

Unlike mice with IL-10 deficiency generated by gene target using homologous recombination (22), we constructed an IL-10 mutant hamster model using CRISPR/Cas9 gene editing due to an unsolved issue of embryonic cell culture *in vitro* (23). Although mRNA expression levels of IL-10 were reduced by 60% in IL10^MUT/MUT^ hamsters, IL-10 protein was largely absent in circulation, indicating that the mutant IL-10 protein produced was not stable. Previous interspecies studies reported that amino acids GLN-LEU-ASP-ASN at positions 42 to 45 are conserved among human, mouse, hamster and other species. These residues are localized at the junctions of helices A and B and required for proper protein folding and maturation (24). Moreover, GLN42 and ASP44 have been identified as essential for the binding of IL-10 to IL-10RA (25, 26). Thus, CRISPR/Cas9-based targeting IL-10 at positions at 42-45 generated a dysfunctional mutant IL-10 protein, effectively silencing IL-10 signaling and causing systemic inflammation.

Patients carrying loss-of-function IL-10 or IL-10R mutations are at a high risk of Ulcerative colitis (UC) and Crohn’s disease (CD), IBD syndromes which involve chronic inflammation of the gut. IBD is characterized by diarrhea, rectal bleeding, abdominal pain, fatigue and weight loss, depending on the severity of inflammation (27). We showed that the lack of IL-10 in hamsters caused an overt reduction in body weight and reduction in WAT mass, similar to that reported in mice (28); however, unlike IL-10 knockout (KO) mice with only mild inflammation, IL-10 mutant hamsters displayed severe systemic inflammation and swelling, suggesting that hamsters were predisposed to active IBD caused by IL-10 deficiency.

Interestingly, Rajbhandari and colleagues reported that loss of IL-10 in mice increased adipose thermogenesis, browning of WAT and improved insulin sensitivity (28). However, the IL-10 KO mice used by Rajbhandari et al. did not exhibit IBD or systemic inflammation. In our hamster model, the expression of thermogenic genes was increased in BAT but not WAT. Histological analysis revealed macrophage accumulation and increased cell death in WAT in IL10^MUT/MUT^ hamsters. These findings suggest that inflammation-mediated induction of apoptosis plays key role in WAT dystrophy in our model. Furthermore, in contrast to the improved insulin sensitivity reported in IL-10 KO mice, we observed impaired glucose and insulin tolerance consistent with the expected consequences of increased systemic inflammation in our model. The basis for the differences in gut and systemic inflammation between IL-10-deficient mice and hamsters not yet clear.

LPS, derived from Gram-negative bacteria in gut (29), is a potent inducer of inflammation. Patients with IBD have been reported to show abnormal gut microbiota profiles (30). We hypothesized that homeostasis of gut microbiota was impaired in IL10^MUT/MUT^ hamsters. Our multi-analysis of microbiota from feces showed an increase in the contents of *Proteobacteria* species and a reduction in *Firmicutes* species, as well as reduced concentration of probiotics such as *lactobacillus* and *allobaculum* in IL10^MUT/MUT^ hamsters. We propose that this impairment of gut microbial homeostasis leads to destruction of gut barrier and enhanced delivery of LPS to the circulation and systemic inflammation (31). Interestingly, our data revealed that although the concentration of LPS in plasma, WAT and liver was elevated in IL-10^MUT/MUT^ hamsters, the co-localization of LPS and macrophages was only seen in WAT, suggesting differential mechanisms regulating the trafficking of LPS. Previously, Hersoug et al. showed that LPS was incorporated into lipoproteins after entering circulation and then internalized by macrophages in WAT (32), whereas LPS/lipoprotein complexes were cleared by the liver through a lipoprotein receptor-mediated pathway (33). Our findings in the hamster model are consistent with these observations.

Lipoproteins play an important role in LPS metabolism (31). Syrian golden hamsters exhibit metabolic traits similar to humans, with cholesterol carried predominant on LDL, ApoB editing only in the intestine, and CETP expression (18). Thus hamsters are an ideal model to better understand the influence of IL-10 on lipoprotein metabolism and atherosclerosis. In hamsters, lack of IL-10 caused reduced TG levels without affecting TC concentration; however, lipoprotein fractionation by FPLC demonstrated that the peak of ApoB-containing LDL was increased, whereas ApoA1-containing HDL was decreased. In IL10^MUT/MUT^ hamsters, we speculate that LPL-mediated lipolysis was activated to release free fatty acids (FFAs), which were delivered to liver for TG synthesis. Enhanced VLDL secretion then led to reduced hepatic TG concentrations.

Given that we observed no changes in plasma or hepatic TC or in expression of genes involved in cholesterol synthesis in mutant hamsters, it is possible that more VLDL was converted to LDL in the setting of inflammation-stimulated lipolysis. Our finding that levels of hepatic LCAT and ABCA1, two key genes required for RCT, were reduced may account for the reduction in HDL levels. Of note, LPS preferably binds to ApoB-containing lipoproteins such as LDL and VLDL (34). In the setting of inflammation caused by IL-10 deficiency, LPS and LDL might form complexes that would be delivered to adipose and liver through the pathways described above.

Dietary lipids have been shown to interact with inflammation to worsen dyslidemia. When challenged with HFD, IL10^MUT/MUT^ hamsters developed an unexpected lipoprotein profile with increased TG and reduced HDL-C, but no changes in TC or LDL-C. In contrast to the reduced TG level seen in CD-fed IL10^MUT/MUT^ hamsters, TG was increased in IL10^MUT/MUT^ hamsters under the HFD condition. This finding may result from dystrophic WAT not being able to properly store dietary lipids. The difference in plasma LDL-C and ApoB levels seen on CD may have been masked by diet-induced cholesterol overload with HFD.

Recently, Johansen and colleagues found in the Copenhagen General Population Study that TG-rich VLDL is more atherogenic than LDL per-particle, implying a higher risk of myocardial infarction (35). These finding suggest that VLDL with more TG and cholesterol in IL10^MUT/MUT^ hamsters was more atherogenic, even though the levels of TC and LDL-C did not change. Interestingly, when analyzing the components of atherosclerotic plaques, we found accumulation of macrophages with LPS, indicating that LDL also participated in the process of disease. LDL has been reported to have a prolonged retention time in the subendothelial space compared to VLDL (35). Our *in vitro* studies revealed that CD36, a molecule responsible for lipid uptake, was increased and LRP1-mediated cholesterol efflux was restrained, ultimately leading to LDL accumulation in IL10^MUT/MUT^ peritoneal macrophages. Therefore, we speculate that VLDL and LDL coordinated with inflammation to accelerate atherosclerosis in IL10^MUT/MUT^ hamsters. Future studies will be needed to clarify the detailed molecular mechanisms by which IL-10 deficiency impairs the LRP1-mediated RCT pathway to promote atherosclerosis.

Direct supplementation of rhIL-10 protein has been shown to alleviate the symptoms of IBD in both IL-10 KO mice and patients with Crohn’s disease (36, 37). In line with these results, we found that administration of rhIL10 for 3 weeks ameliorated inflammation and improved body weight and dyslipidemia in IL10^MUT/MUT^ hamsters. However, because half-life of IL-10 in circulation is only 4.5 hours, daily repeated injections were required to maintain IL-10 at a physiological level in these experiments. Patients with IBD receiving purified IL-10 have adverse effects such as hemoglobin reduction or thrombocytopenia, limiting its therapeutic utility. Emerging data have demonstrated that the adeno-associated virus (AAV) is a promising approach to deliver gene therapy for human disease (38). It will be of interest to use AAV-mediated gene transfer to treat IL10^MUT/MUT^ hamsters as a prelude to considering IL-10 gene therapy for IBD.

In conclusion, we report an IL-10 mutant hamster model and characterize the phenotypes caused by IL-10 deficiency. Our findings clearly demonstrate a role for IL-10 in maintaining lipid and tissue homeostasis, and in preventing atherosclerotic lesion development. These results confirm the causal relationship between IL-10 and atherosclerosis and providing new insight into IL-10 as a potential therapeutic for the prevention or treatment of atherosclerosis.

## Data availability statement

The datasets presented in this study can be found in the repository PRIDE with the accession number PXD036331.

## Ethics statement

The animal study was reviewed and approved by Peking University.

## Author contributions

XX conceived and designed the study. HS, JG, QY, MG, LW, and LZ performed the experiments. HS and JG wrote the original manuscript. HS, JG, XH, LL, YW, WH, and XX interpreted the data. YW, GL and XX acquired the funding. GL and XX supervised the study. PT and XX reviewed and edited the manuscript. All authors contributed to the article and approved the submitted version.

## Acknowledgments

This work was supported by National Natural Science Foundation of China (NSFC) 31520103909 and 91739105 to GL, 81770449 to YW, 81860224 to LW and 82070460 to XX. GL is a fellow at the Collaborative Innovation Center for Cardiovascular Disease Translational Medicine, Nanjing Medical University.

## Conflict of interest

The authors declare that the research was conducted in the absence of any commercial or financial relationships that could be construed as a potential conflict of interest.

## Publisher’s note

All claims expressed in this article are solely those of the authors and do not necessarily represent those of their affiliated organizations, or those of the publisher, the editors and the reviewers. Any product that may be evaluated in this article, or claim that may be made by its manufacturer, is not guaranteed or endorsed by the publisher.
