# Supplementary material for: CRISPR/Cas9 based blockade of IL-10 signaling impairs lipid and tissue homeostasis to accelerate atherosclerosis
Source: Front Immunol. 2022 Aug 30;13:999470. doi: 10.3389/fimmu.2022.999470 (PMC9469689; doi:10.3389/fimmu.2022.999470)
Supplement: Supplementary file 1 [file DataSheet_1.pdf]

Supplementary figure legends

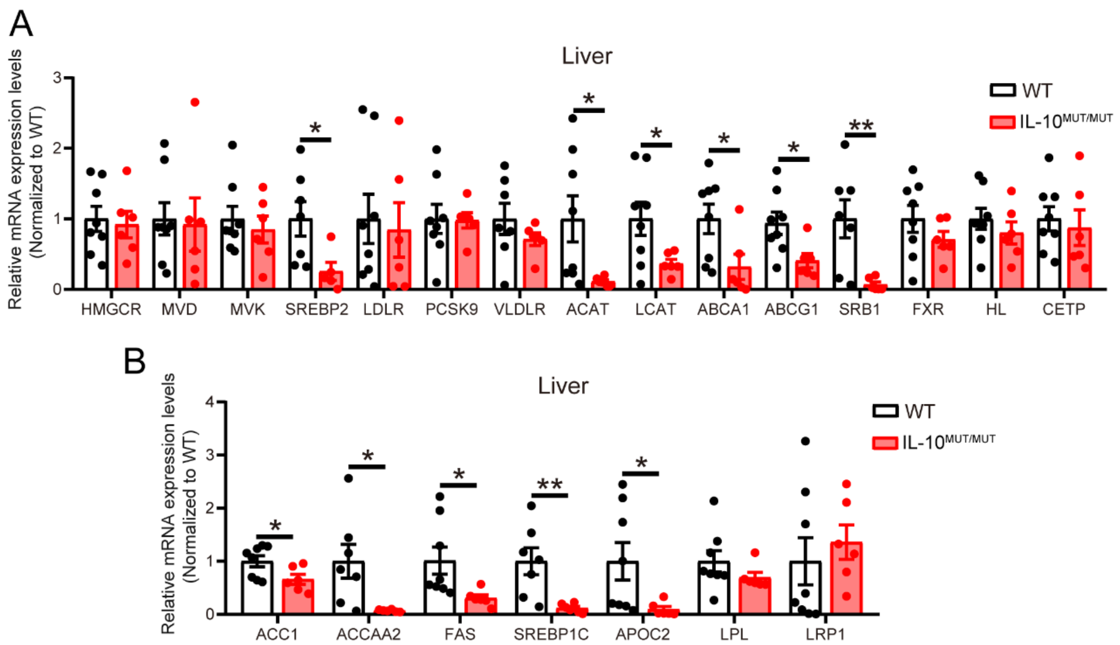

Figure S1. Chow diet-fed IL10$^{MUT/MUT}$ hamsters showed abnormal fatty acid and cholesterol metabolism in liver.

A: Expression levels of genes involved in cholesterol metabolism were determined by real-time PCR (n=6 ~ 8/group).

B: Expression levels of genes involved in fatty acid metabolism were determined by real-time PCR (n=6 ~ 8/group).

All date were expressed as means ± SEM, *, $p<0.05$; **, $p<0.01$.

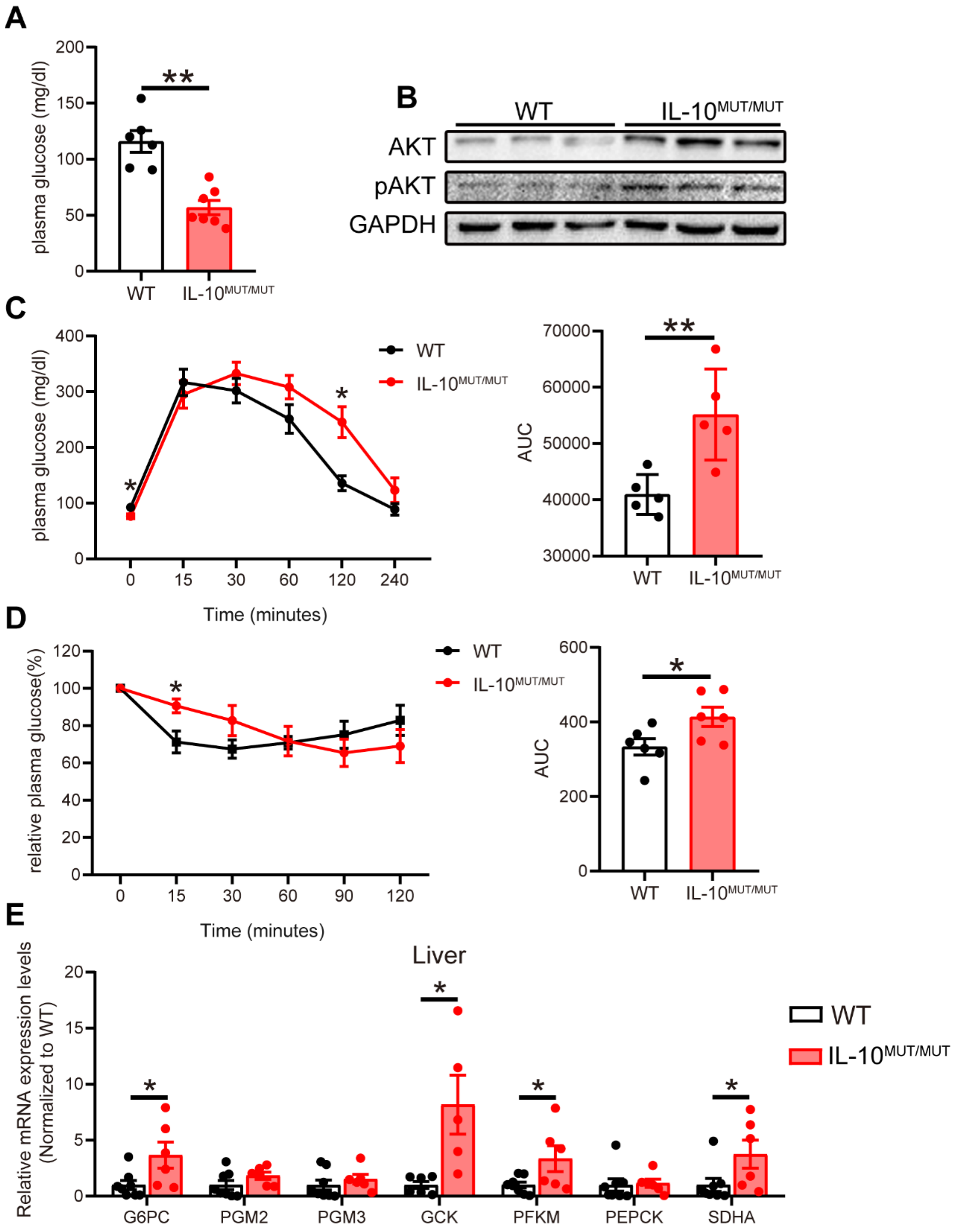

Figure S2. Chow diet-fed IL10$^{MUT/MUT}$ hamsters showed abnormal glucose metabolism.

A: Blood glucose levels were determined from WT and IL10$^{MUT/MUT}$ hamsters on CD (n=6~7/group).

B: Representative Western blot of hepatic AKT and pAKT from WT and IL10$^{MUT/MUT}$ hamsters.

C, D: GTT (C) and ITT (D) was performed on WT and IL10$^{MUT/MUT}$ hamsters fed CD. Plasma glucose curves were shown in left panel and quantification by AUC was shown in right panel (n=5~6/group).

E: Expression levels of genes regulating gluconeogenesis in liver were determined by real-time PCR (n=5~7/group).

All date were expressed as means ± SEM, *, $p<0.05$; **, $p<0.01$.

LOCUS	gene name	primer sequence
NW_024429191.1	Igfbp4	F: GGGCTGTAGAGTTGACCTGA<br>R: TCTTCCACACAAAGCTCCCTC
NW_024429203.1	Atm	F: ACTCAGGGGTTAGGAGATACACT<br>R: TCTGCGTGATGTAGACTGCT
NW_024429195.1	Agap2	F: GTTGAGGAGTTCTGGGGCAC<br>R: GTAGTAATGCCCCCTCCACG
NW_024429184.1	Rab27b	F: GAGTGCCTGTGAAGGAGACC<br>R: CCCTGTTGCCTGAAGACCAA
NW_024429266.1	B3galt2	F: GGGAGGAAAAGGGATACAGGT<br>R: TGGGGCCAAACAAAACATGAG

Table. S1 The list of potential off-target genes and the PCR primers for analysis in founder.

Gene	Forward	Reverse
Beta actin	ACTGCCGCATCCTCTTCCT	TCGTTGCCAATGGTGATGAC
IL-10	ACCTGGTAGAAGTGATGCCC	AGTTGCCTCCTGAGGGTCTT
IL-10RA	CCCAGGGCTACCAGAAACAG	GACCCTTGATTCTGGGGGTG
IL-10RB	TCAACGAAGAGACCGAAGGC	CGAGGCCGACGTAAGAAGTA
STAT1	GGACGGCAAAGTCAGAAACG	TGTCTTCGCTTCCACTCCAC
STAT3	GCCAAATGCATGGGCATCAA	TTGGTCCCAGGTTCCAATCG
JAK1	GTACGCTCCGAACCACATCA	CCGCCATACAGACTGCTCAT
TYK2	GCCCTGGTGGATGGCTATTT	GGATGCCGTTCTGGATGCTA
IL-1β	AGTCATTGTGGCTGTGGAGA	TGTTGTTCATCTCGGAGCCT
IL-6	CAACCCTGGCTGTATGGACA	GTGCTCTGAATGACTCTGGCT
NFκB	ATGAAGACTTGGGGGCCTTG	CTGTGGGGTGAGACATGGAC
TNFα	TCCTGGCCTCCTTTTTGCTT	CCCGTAGGGCGATTACAGTC
UCP-1	TTAATGACCGGAGGTGTGGC	CCTCAACAGGTTAGGGGTCG
CIDEA	GGACAGTTCCTGGTCTACGC	AAAGGAATGCACCTGGGCTC
COX8B	AGTTCCCCAGGCGGCTATAA	AGGTTGTGCTCCTTCCTTGG
PGC1A	TGAATGCAGCGGTCTTAGCA	TTGGAGGCGCATTTGTCTCT
BAX	GGCCTTTTTGCTACAGGGTTTC	TCATCTCCGATTCGCCTGAG
BCL2	AAATCGCCGAGAAGAAGCGA	GTTCCACGGTTTGGCTTCAC
MLKL	TGAAGATTGCCCCAAGCTGT	TCCAGTTTGTTCCGCTTCCA
MYD88	ATTGAGAAGAGGTGTCGCCG	GATGGTGATGAACCGCAGGA
TLR4	GCTGAAGCAACAAGTGGAGC	GGCTCTTCCGTCCAACAGAG
LBP	ATCTGGGTCCGAGGCAAATG	GGAGCTTGGACTCGATCTGG
CD14	TCCCGGCTCTCCAAGTTCTA	CGGTATGCCGCAGTGAATTG
ABHD5	CGGATAGGAGACTTGCACCC	TCACGTAGGACTTTGGTCGC
ATSL	AAGGAGTGCGCTATGTGGAC	GATTGCGCAGGTTGAACTGG
HSL	GTTGTCGTCCCTGGCTAACA	TTCCCGCAGGTCATAGGAGA
PLIN1	CCCAGCCCTTCAATACCCTC	TGGTGTGCCGAGAAAGAGTG
LRP1	TGTACGCCACCAACTCAGAC	CTTCTTGCAGGACTTCCCGT

SHC1	TGGTTCGAGAGAGCACAACC	CCTTTGTCCGAACCACACCT
LXRα	GTCCACAAAAGCGGAAAAAG	CTCGCAGCTCAGAACAATGTA
ABCA1	TTGGATGGATTAGATTGGACTGC	TGGTTTCATTGAAAGCTTCTCTC
ABCG1	GAGACAGACCTGCTGAATGGG	CAGTCTCTCTGTATCCCGCC
CD36	GAGAAGTCTCGGGTACTGCG	TTGTCTGGATTCTGGAGCGG
SRB1	GAGCCCCTGAACACGTTCTAT	CTGTCCGCTGAGAGAGTCCT
G6PC	GTCCCGGATCTACCTTGCAG	ATTCTGGCCGTTCACACCAT
PGM2	CTCGCTCCTCCTGAAGCTGT	TTGGCCTTGATCTTGTCAGC
PGM3	AACGGCATAGGAGCCTTGAA	CAGCACCACACAGGTGATTG
GCK	ACCTTGCCTGGGGAAATAGC	CATGTGGCTGGACAGTCAGT
PFKM	ACGGAGCTGAAGGACCAGAC	GCGTGGTCAGAAGTGTCCAA
PEPCK	GACAGTGCCCAAGAGACTCC	TTGGGCAGATCCTGGTTGAC
SDHA	TGGCGTGGATGTCACTAAGG	GCACAACCTGATCCTGTCCA
HMGCR	TGTTCATGCTCACAGTCGCT	AGCTCTGCTTGTGCTCTCAG
MVD	CTGCCTGAGGGAGATTCGAC	TCACCACGAGGATGAGGACT
MVK	TGCTTCGACCACAGAGCAAT	TTGGTGCAGACATGGTCCTC
SREBP2	AGTTGGCAAACCAAAAAAACAAG	GATTAAAGTCTTCAATCTTCAAGTCCAC
LDLR	GCATCACACTAGATATTCCCAGT	GAGTTTGGAATCAACCCAATAGA
PCSK9	CCAACTTTGGACGCTGTGTG	TGAAGCATGCGCTACAGTCA
VLDLR	CAGAACAGTGCCGCAACATC	ACAGTCCAGCTCATCACTGC
ACAT	CCAGCACACTGAACGATGGA	AGGTACAGCATATGCAGGCG
LCAT	TCCCTAAGGCTATGCCCCTT	AGCACAACCAGTTCACCACA
FXR	TCTTCAGGAGAAGCATTACCAA	CGCATGTACATATCCATCACAC
HL	CCTAATCGGCTACAGCCTGG	GTCATAGTGGGCAATGGGCT
CETP	AAGCCTTGCGGATTTCCAGA	CACGACTGATGATCTCGGGG
ACC1	ACACTGGCTGGCTGGACAG	CACACAACTCCCAACATGGTG
ACCAA2	GAAGCTTTACCATGGCGCTG	TCACGATGGACTGGAAACCG
FAS	GCAGTCTTGAGTAGCTTTGTGCT	GGGAGCTGTCCAGATTAATACCT
SREBP1C	GCGGACGCAGTCTGGG	ATGAGCTGGAGCATGTCTTCAAA

APOC2	AAGCTCAAGGAACGGAGGAC	GCTGGTCGGTAAAAATGCCC

Table. S2 The list of primers used for quantitative real time PCR.
